# *GPR174 *mRNA Acts as a Novel Prognostic Biomarker for Patients With Sepsis *via* Regulating the Inflammatory Response

**DOI:** 10.3389/fimmu.2021.789141

**Published:** 2022-01-31

**Authors:** Jianli Wang, Yanyan Hu, Zhongshu Kuang, Yao Chen, Lingyu Xing, Wei Wei, Mingming Xue, Sucheng Mu, Chaoyang Tong, Yilin Yang, Zhenju Song

**Affiliations:** ^1^ Department of Emergency Medicine, Zhongshan Hospital, Fudan University, Shanghai, China; ^2^ Shanghai Key Laboratory of Lung Inflammation and Injury, Shanghai, China; ^3^ Shanghai Institute of Infectious Disease and Biosecurity, School of Public Health, Fudan University, Shanghai, China

**Keywords:** GPR174, sepsis, biomarker, prognosis, immune response

## Abstract

Previous studies indicated that G-protein coupled receptor 174 (GPR174) is involved in the dysregulated immune response of sepsis, however, the clinical value and effects of GPR174 in septic patients are still unknown. This study is aimed to evaluate the potential value of GPR174 as a prognostic biomarker for sepsis and explore the pathological function of GPR174 in cecal ligation and puncture (CLP)-induced septic mice. In this prospective longitudinal study, the expressions of peripheral *GPR174* mRNA were measured in 101 septic patients, 104 non-septic ICU controls, and 46 healthy volunteers at Day 1, 7 after ICU (Intensive Care Unit) admission, respectively. Then, the clinical values of GPR174 for the diagnosis, severity assessment, and prognosis of sepsis were analyzed. Moreover, the expressions of *GPR174* mRNA in CLP-induced septic mice were detected, and *Gpr174*-knockout (KO) mice were used to explore its effects on inflammation. The results showed that the levels of *GPR174* mRNA were significantly decreased in septic patients compared with non-septic ICU and healthy controls. In addition, the expressions of *GPR174* mRNA were correlated with the lymphocyte (Lym) counts, C-reactive protein (CRP), and APACHE II and SOFA scores. The levels of *GPR174* mRNA at Day 7 had a high AUC in predicting the death of sepsis (0.83). Further, we divided the septic patients into the higher and lower *GPR174* mRNA expression groups by the ROC cut-off point, and the lower group was significantly associated with poor survival rate (P = 0.00139). Similarly, the expressions of peripheral *Gpr174* mRNA in CLP-induced septic mice were also significantly decreased, and recovered after 72 h. Intriguingly, *Gpr174*-deficient could successfully improve the outcome with less multi-organ damage, which was mainly due to an increased level of IL-10, and decreased levels of IL-1β and TNF-α. Further, RNA-seq showed that *Gpr174* deficiency significantly induced a phenotypic shift toward multiple immune response pathways in septic mice. In summary, our results indicated that the expressions of *GPR174* mRNA were associated with the severity of sepsis, suggesting that GPR174 could be a potential prognosis biomarker for sepsis. In addition, GPR174 plays an important role in the development of sepsis by regulating the inflammatory response.

## Introduction

Sepsis is a life-threatening organ dysfunction that is caused by a dysregulated host response to infection (Sepsis-3) (1). In 2017, a WHO resolution recognized sepsis as a global health priority with an unacceptably high mortality rate of 30 to 50% (2). It is well known that systemic inflammatory response syndrome accompanied by a hyper-inflammatory state leads to multiple organ dysfunction syndromes (MODS) or death (3). With further studies of immune response and pathological mechanism of sepsis, several markers have been found and evaluated for early recognition, diagnosis, and management of sepsis (4–6). However, finding novel diagnostic and targeted therapeutic biomarkers is still pivotal for septic patients due to the unsatisfactory specificity and sensitivity of the currently available biomarkers (7).

G protein-coupled receptors (GPCRs) are a group of cell surface receptors that detect extracellular molecules and activate cellular responses (8). GPCRs have been considered as one of the largest families of validated drug targets, which involve almost overall physiological functions and pathological processes. Approximately 34% of Food and Drug Administration (FDA)-approved drugs target this family (9). GPR174 is activated by lysophosphatidylserine (LysoPS), a lipid mediator known to induce rapid degranulation of mast cells, suppress proliferation of T lymphocytes, and enhance macrophage phagocytosis of apoptotic neutrophils (10, 11). GPR174 has been reported to play an important role in regulating the functions of regulatory T cells and activating B cells (12, 13). *In vitro*, overexpressed GPR174 effectively inhibited the proliferation of CHO cells stimulated by LysoPS (14). In a mice model of experimental autoimmune encephalomyelitis (EAE), the deficiency of *Gpr174* resulted in immunological tolerance to IFN-γ-dependent lesion by constraining regulatory T cells’ development and function (15). In angiotensin II (Ang II)-treated mice, GPR174 could inhibit retinopathy by reducing inflammation (16). In addition, a genome-wide association study (GWAS) in the Chinese population implied that GPR174 variation could be a risk factor for Graves’s disease (17).

Our previous studies indicated that lack of *Gpr174* significantly decreased the concentrations of proinflammatory cytokines, such as tumor necrosis factor-α (TNF-α), interleukin-1β (IL-1β) in lipopolysaccharide (LPS)-induced septic mice (18), which suggested that GPR174 might be considered as a potential biomarker for the patients with sepsis. However, the clinical values of GPR174 for the diagnosis, severity, and prognosis of sepsis have yet to be investigated. Thus, we conducted a prospective, non-interventional cohort study to assess the association between the levels of *GPR174* mRNA and the severity and mortality of septic patients. Further, we explored the potential effects and mechanism of GPR174 on the host immune responses in CLP-induced mice.

## Materials and Methods

### Septic Patients and Controls

A prospective study was carried out in the emergency intensive care unit (ICU) of Zhongshan Hospital, Fudan University, Shanghai, China. From December 2017 to September 2019, 101 septic patients who met the clinical criteria for sepsis-3 were enrolled (1). There were 104 non-septic patients in the ICU (poly-trauma, cerebral trauma, intracranial hemorrhage, cerebrovascular accidents, and hypertensive emergencies) recruited as non-septic ICU controls. Furthermore, septic patients were divided into survival group and non-survival group according to the survival of 90 days.

Exclusion criteria included age below 18 years, pregnancy, severe chronic respiratory disease, severe chronic liver disease (defined as Child-Pugh score > 10), malignancy, immune disease, using high-dose immunosuppressive therapy, and AIDS patients. In addition, 46 age- and gender-matched healthy volunteers with no medical problems were obtained from the medical examination center of Zhongshan Hospital, Fudan University, China. The flowchart of this study is shown in **Supplementary Figure 1**.

Patients’ characteristics (age, gender, and previous health status), as well as clinical data including Sequential Organ Failure Assessment (SOFA) and Acute Physiology and Chronic Health Evaluation II (APACHE II) scores, source of primary infection, and ICU mortality, were obtained after ICU admission. The characteristics of patients, mechanical ventilation, and vasopressor treatment are shown in **Table 1**. This study was approved by the Ethics Committee Study Board of Zhongshan Hospital, Fudan University, Shanghai, China (number: B2014-082). Written informed consent was obtained from patients, the next of kin, or guardians on the behalf of the participants before enrollment according to the Declaration of Helsinki.

**Table 1:** Baseline characteristics of patients at ICU admission.

Variables	Non-septic ICU Controls	Septic Patients	*P*. value
n	104	101	
Demographics			
Age, year, mean ± S.D.	61.6 ± 18.9	61.5 ± 16.7	0.937
Male, sex, n (%)	58 (55.8)	55 (54.5)	0.85
Patients’ outcomes			
90-d mortality, n (%)	2 (1.9)	26 (25.7)	<0.001
Severity of disease			
SOFA score, median (IQR)	1 (0 - 3)	4 (2 - 7)	<0.001
APACHE II score, media (IQR)	7 (4 - 11)	12 (7 - 19)	<0.001
Primary site of infections			
Pneumonia, n (%)		62 (61.4)	
Urinary tract infections, n (%)		10 (9.9)	
Intra-abdominal infections, n (%)		18 (17.8)	
Blood, n (%)		8 (7.9)	
Others, n (%)		3 (3.0)	
Invasive ventilation, n (%)	3 (2.9)	30 (29.7)	<0.001
Non-invasive ventilation, n (%)	6 (5.7)	23 (22.8)	<0.001
CRRT, n (%)	0 (0)	2 (1.98)	0.143
Vasopressors, n (%)	1 (0.96)	22 (27)	<0.001
Laboratory parameters			
CRP, mg/L, median (IQR)	47.8 (17.2, 100.1)	86.9 (26.1, 150.4)	0.010
PCT, ng/mL, median (IQR)	0.2 (0.1, 1.3)	0.8 (0.2, 8.9)	<0.001
WBC, *10^9^/L, median (IQR)	8.4 (6.0, 11.5)	10.8 (7.1, 15.5)	0.001
Neu, *10^9^/L, median (IQR)	6.3 (3.9, 9.3)	9.3 (5.7, 13.5)	<0.001
Lym, *10^9^/L, median (IQR)	1.1 (0.8, 1.7)	0.9 (0.5, 1.3)	<0.001
IL-2R, U/mL, median (IQR)	842 (530, 1325)	1116 (694, 1851)	0.001
IL-6, pg/mL, median (IQR)	16.1 (7.8, 52.1)	29.8 (10.2, 89.1)	0.015
IL-8, pg/mL, median (IQR)	21.0 (13.3, 40.0)	44.0 (24.0, 82.0)	<0.001
IL-10, pg/mL, median (IQR)	5.0 (5.0, 8.5)	8.6 (5.0, 17.8)	<0.001

ICU, Intensive Care Unit; SOFA, sequential organ failure assessment; APACHE, acute physiology and chronic health evaluation; CRP, C-reactive protein; PCT, procalcitonin; WBC, white blood cells; Neu, neutrophil; Lym, Lymphocyte.

### Mice

*Gpr174* knock-out (*Gpr174*-KO) mice generated by a homologous recombination method were provided by Shanghai Southern Model Biotechnology Co. Ltd. (Shanghai, China). Age- and gender-matched C57BL/6 mice were obtained from Fudan University, Shanghai, China. Mice were housed under a specific pathogen-free condition with a 12 h-light/12 h-dark cycle, 22 ± 2°C. Animal experiments were approved by the Ethics Committee of Laboratory Animal Science, Fudan University, China (number 201804001Z).

### CLP-Induced Septic Mouse Model

The CLP-induced septic mouse model was established as described previously (19). In short, mice were anesthetized with 1% pentobarbital (i.p. 10 ml/kg of body weight). After that, the cecum was ligated in half, and a 21-gauge needle was used to puncture the stump once to squeeze out a small number of feces. The cecum was placed back into a normal intraabdominal position, and the abdominal incision was closed by applying sample running sutures. Then, pre-warmed normal saline (50 ml/kg of body weight) was injected subcutaneously. Sham-operated control mice underwent the same surgical procedures without puncture or ligation. The survival rate was monitored daily for 1 week.

### Quantitative Polymerase Chain Reaction

The expressions of *GPR174* mRNA in peripheral blood of all samples were tested by quantitative PCR on Day 1 (D1) and Day 7 (D7) after ICU admission. Peripheral blood mononuclear cells (PBMC) were isolated from fresh anticoagulant blood by Ficoll lymphocyte separation solution (Lymphoprep, Axis-Shield, UK). The total RNAs from all enrolled subjects and mice were extracted using TRIzol reagent (Life Technologies, Carlsbad, CA) and then 10 μg of RNA samples were reverse-transcribed into cDNA with Prime Script™ reagent kit (Takara, Dalian, China) following the manufacturer’s instructions. Quantitative PCR was performed with SYBR^®^ Premix Ex Taq™ II (Takara, Dalian, China) on a 7500 Real-time PCR system (Applied Biosystems, Carlsbad, CA), according to the manufacturer’s instructions.

Primers used were as follows:

human-GPR174 sense 5’-ATCATCTGCCTTGCCTGTGTACTC, antisense 5’-CGCCAATGGTCATCATAACAACGG; human-GAPDH sense 5’-AAGGTCGGAGTCAACGGATT, antisense 5’- CTCCTGGAAGATGGTGATGG; mouse-Gpr174 sense 5’- TTGGTCTGCATCAGTGTGCGAAG, antisense 5’-CAGGCAGGCAAGGCAGATGATC; mouse-Gapdh sense 5’-GGAGAGTGTTTCCTCGTCCC, antisense 5’-ACTGTGCCGTTGAATTTGCC; mouse-Cfh sense; 5’- GTATCAAAACGGATTGTGACGT, antisense 5’- TAACACATGTCACAGTGTCTGA; mouse-Oas1g sense 5’- TAAGAAACAGCTGTACGAGGTT, antisense 5’- CCAGATGAGGATGGTGTAGATT; mouse-Spp1 sense 5’- AAACACACAGACTTGAGCATTC, antisense 5’- TTAGGGTCTAGGACTAGCTTGT.

### Cytokine’s Measurement

Blood samples of septic and ICU non-septic patients and mice were centrifuged (3500 rpm, 15 min, room temperature) after collection. The serum was immediately frozen in liquid nitrogen (LN2) and stored at -80°C for further use. The levels of IL-1β, IL-2R, IL-6, IL-8, and IL-10 were measured by ELISA kit (R&D Systems, Inc., Minneapolis, MN) according to the manufacturer’s protocol. Serum concentrations of CRP and PCT were measured by IMMAGE800 analyzer (Beckman Coulter, Inc. CA) and VIDAS B.R.A.H.M.S PCT analyzer (Biomerieux, Lyon, France). Routine blood tests and blood gas analysis were conducted in the clinical laboratory of Zhongshan Hospital, Fudan University, Shanghai, China.

### Hematoxylin and Eosin Stain

Tissues (lung, liver) were excised at 0 h, 12 h, 24 h, 72 h, and 1 and 2 weeks after CLP, washed with DPBS, fixed with 4% formalin buffer, and paraffin embedded. There were 4-6 μm sections cut and placed on glass slides, deparaffinized in xylene, and rehydrated in a series of alcohol solutions. Sections were then washed in distilled water, and stained with H&E for histopathological examination.

### RNA-Sequencing

Total RNA was digested by DNase I (Qiagene) and separated by Dynabeads^®^ mRNA DIRECT™ Kit (Life Technologies). The isolated mRNAs were used for mRNA-seq libraries with a KAPA Stranded mRNA-Seq Kit according to the manufacturer’s instructions. Libraries were sequenced on the Hiseq Xten system (Illumina) with a reading length of 150 base pairs (bps). Differentially expressed genes (DEGs) between *Gpr174*-KO and Wild type (WT) mice were identified using the Bioconductor package RUVSeq (version 1.0.0, http://www.bioconductor.org). Hierarchical cluster analysis of the DEGs was carried out by the “hclust” function of the “stats” package in R software (https://www.r-project.org/). The heatmap for this cluster analysis of the DE genes was drawn with heatmap.2 function in the “gplots” package. Gene ontology analysis (GO analysis) performed by Blast2GO software (version 4) was used to provide a further understanding of these results.

### Statistical Analysis

Normal distribution data were expressed as means and standard deviations (S.D.) with Student’s t-test or ANOVA test. Non-normal distribution continuous data were expressed as medians with the 25th and 75th quartiles applying Mann-Whitney U test or Kruskal-Wallis test followed by Dunn’s multiple comparisons post-test. Categorical data were expressed as frequency and percentage. Non-parametric Spearman’s rank correlation coefficient was performed to test correlations between two parameters. For analyzing the independent predictors of 90-day mortality, binary logistic regression was used to determine the discriminative power of *GPR174* mRNA for 90-day mortality. Receiver-operating characteristic (ROC) curves were constructed and the area under the curve (AUC) was determined with a 95% confidence interval (CI). The bootstrap and Venkatraman’s test were used for comparing the AUC using the “pROC” package in R (20). Kaplan-Meier curves of disease-free survival were plotted and compared by Cox regression analysis in the groups layering by ROC cut-off point. For murine survival studies, Kaplan-Meier analyses followed by log-rank tests were performed. All statistical analyses were done using SPSS 16.0, R 4.0.2, and GraphPad Prism version 5.01 (GraphPad Software, San Diego, CA). P values less than 0.05 were considered statistically significant.

## Results

### Baseline Characteristics of Septic and Non-Septic ICU Patients

The demographic and clinical characteristics of septic patients and non-septic ICU controls are presented in **Table 1**. No age effects were observed between septic patients and non-septic ICU controls (61.5 vs. 61.6, *P*=0.937). The 90-day mortality was 25.7% in septic patients, while 2% in non-septic ICU controls. After ICU admission (Day 1), septic patients had significantly higher APACHE II and SOFA scores than non-septic ICU controls. Non-survival septic patients also had significantly higher APACHE II and SOFA scores, and the utilization rate of mechanical ventilation and vasoactive agents than survivors (**Table 2**). The levels of serum PCT, IL-2R, IL-6, and the counts of WBC, neutrophil, and lymphocyte were also significantly higher in septic patients than that in non-septic ICU controls (**Table 1**). However, there was no difference between non-survivors and survivors in sepsis patients at Day 1 (D1) for these inflammatory markers (**Table 2**).

**Table 2:** Baseline characteristics of survivors and non-survivors in septic patients at ICU admission.

Parameter	Survivors	Non-survivors	*P* value
*n*	75	26	
Demographics			
Age, year, mean ± S.D.	60 ± 2.03	65 ± 2.94	0.182
Male, sex, *n*	37 (49.3)	18 (69.2)	0.11
Severity of disease			
SOFA score, median (IQR)	2 (1, 4)	7 (4, 9)	0.001
APACHE II score, media (IQR)	7 (5, 13)	16 (13, 24)	<0.001
Primary site of infections			
Pneumonia, *n* (%)	37 (49.3)	25 (96.1)	<0.001
Urinary tract infections, *n* (%)	10 (13.3)		<0.001
Intra-abdominal infections, *n* (%)	18 (24)		<0.001
Blood, *n* (%)	8 (10.6)		<0.001
Others, *n* (%)	2 (2.7)	1 (3.8)	0.093
Invasive ventilation, *n* (%)	12 (16)	18 (69)	<0.001
Non-invasive ventilation, *n* (%)	17 (22.7)	6 (23.1)	0.999
CRRT, *n* (%)	0	2 (7.7)	0.064
Vasopressors, *n* (%)	9 (12)	13 (50)	<0.001
Laboratory parameters			
CRP, mg/L, median (IQR)	55.5 (18.3, 114.5)	90 (31.8, 133.9)	0.699
PCT, ng/mL, median (IQR).	0.4 (0.1, 2.3)	1.4 (0.2, 9.9)	0.643
WBC, *10^9^/L, median (IQR)	8.8 (6.5, 12.5)	12.0 (9.1, 20.0)	0.322
Neu, *10^9^/L, median (IQR)	6.8 (4.5, 10.3)	10.2 (6.8, 17.8)	0.184
Lym, *10^9^/L, median (IQR)	1.0 (0.7, 1.5)	0.6 (0.4, 1.2)	0.104
IL-2R, U/mL, median (IQR)	938 (571, 1483)	1059 (854. 2166)	0.732
IL-6, pg/mL, median (IQR)	21.0 (8.1, 66.0)	47.0 (14.3, 129.0)	0.559
IL-8, pg/mL, median (IQR)	27.0 (15.0, 57.8)	62.0 (22.0, 87.0)	0.473
IL-10, pg/mL, median (IQR)	5.7 (5.0, 11.6)	7.8 (5.4, 12.5)	0.573

ICU, Intensive Care Unit; SOFA, sequential organ failure assessment; APACHE, acute physiology and chronic health evaluation; CRP, C-reactive protein; PCT, procalcitonin; WBC, white blood cells; Neu, neutrophil; Lym, Lymphocyte.

### The Levels of Relative Expressions of *GPR174* mRNA in Septic and Non-Septic ICU Patients

**Figure 1:**
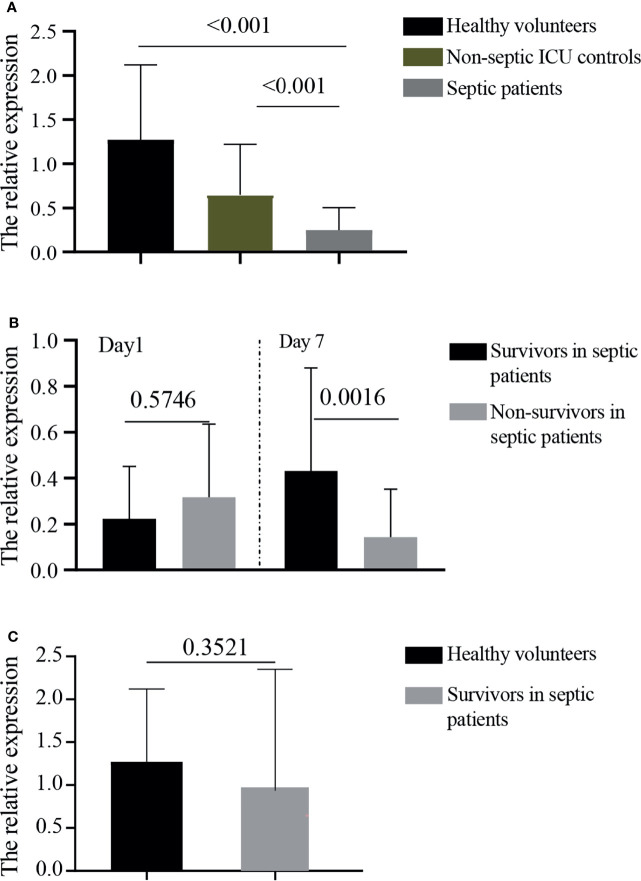
The relative expressions of GPR174 mRNA in patients. **(A)** The concentrations of serum *GPR174* mRNA were measured by quantitative PCR from 101 septic patients, 104 non-septic ICU controls, and 46 healthy volunteers. **(B)** The concentrations of *GPR174* mRNA were measured from survivors and non-survivors in septic patients at Day 1 and Day 7 after ICU admission. **(C)** The levels of *GPR174* mRNA in sepsis survivors at discharge were compared with healthy volunteers. *P* values less than 0.05 were considered statistically significant.

The relative expressions of *GPR174* mRNA in septic patients at D1 were significantly lower than that in non-septic ICU controls and healthy volunteers (**Figure 1A**). The levels of *GPR174* mRNA had no significant difference between survivors and non-survivors in patients with sepsis at D1 (**Figure 1B**). However, lower expressions of serum *GPR174* mRNA were observed in non-survivors compared to survivors at Day 7 after admission (D7) (**Figure 1B**). Interestingly, with the recovery of sepsis, the concentration of *GPR174* mRNA in septic patients returned to the level of healthy subjects (**Figure 1C**).

### The Correlations of *GPR174* mRNA With APACHE II and SOFA Scores, and Inflammatory Cytokines in Septic Patients

**Figure 2:**
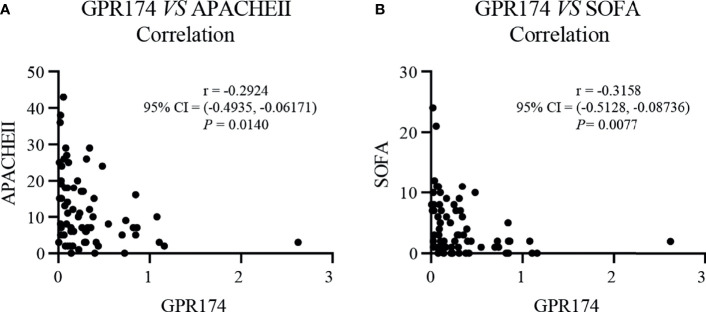
The levels of *GPR174* mRNA were correlated with disease severity in septic patients. **(A)** Correlation of the relative expression of *GPR174* mRNA with APACHE II score in patients with sepsis. **(B)** Correlation of the expression of *GPR174* mRNA with SOFA score in patients with sepsis. Dots represent individual participants. *P* values less than 0.05 were considered statistically significant.

**Figure 3:**
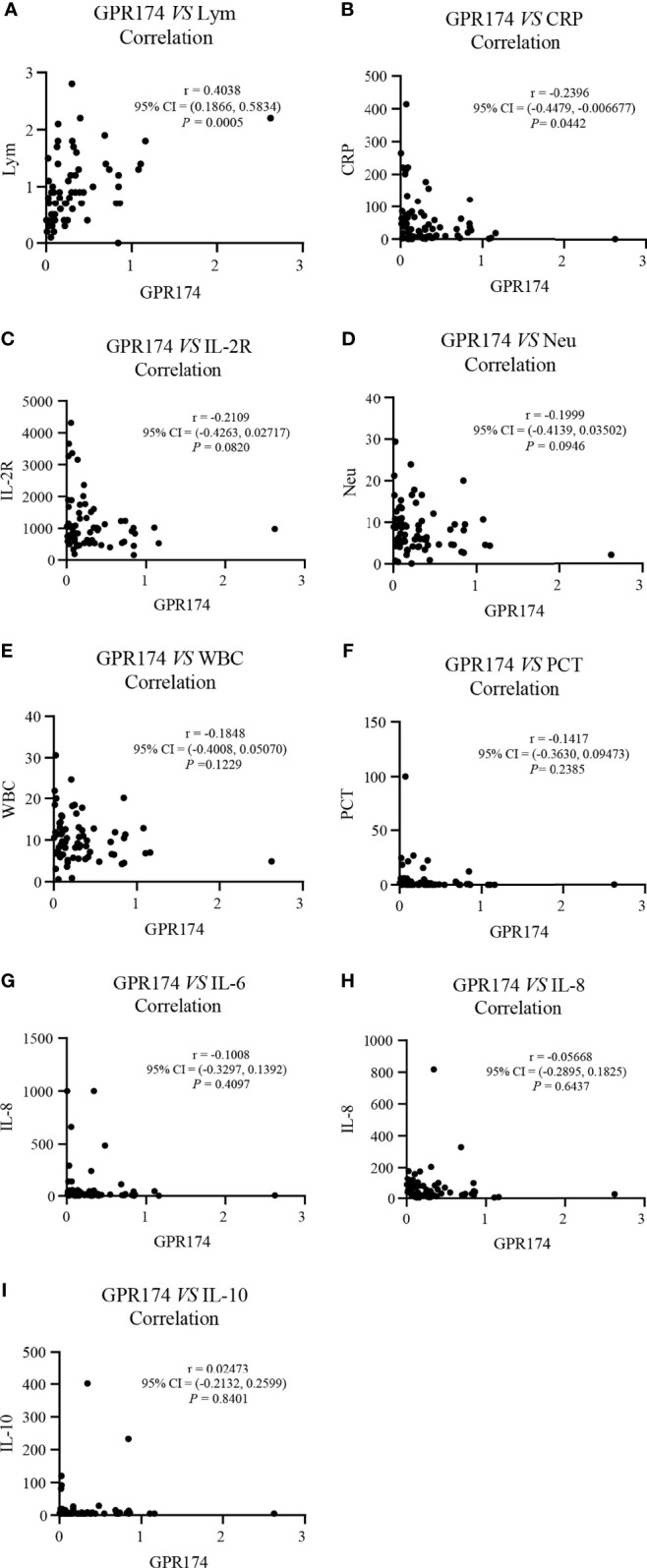
The expression of *GPR174* mRNA correlated with laboratory parameters in septic patients. **(A–I)** Correlation of the expression of *GPR174* mRNA with the counts of WBC, Neu, and Lym, CRP, IL-2R, PCT, IL-6, IL-8, and IL-10 in patients with sepsis, respectively. Dots represent individual participants. *P* values less than 0.05 were considered statistically significant.

Our results showed that *GPR174* mRNA levels were significant negative correlations with APACHE II and SOFA scores (**Figure 2**). As for other biomarkers, a significant negative correlation was observed between *GPR174* mRNA and CRP, while there is a positive correlation for the count of Lym at D7 (**Figure 3**). However, no significant correlations were found among the levels of *GPR174* mRNA and PCT, IL-2R, IL-6, IL8, IL-10, and the count of Neu, WBC at D7.

Then, we detected the individual change of those markers from D1 to D7 both in survivors and non-survivors in sepsis. Interestingly, *GPR174* mRNA showed remarkable differences both in the non-survivor and survivor groups (ascending in the survivors and descending in non-survivors, respectively), while other biomarkers did not show such results, even for APACHE II and SOFA scores (**Supplementary Figure 2**).

### The Correlations of GPR*174* mRNA With 90-Day Mortality in Patients With Sepsis

Using binary logistic regression analysis adjusted by age and gender, both *GPR174* mRNA (OR = 0.004, *P* = 0.003) and CRP (OR = 1.010, *P* = 0.031) at D7 were found to be independent predictors of 90-day mortality in patients with sepsis (**Table 3**).

**Table 3:** Independent factors predicting 90-day mortality in septic patients.

Variable	B	OR	95% CI	*P* value
On the day of admission (Day1)				
GPR174	1.321	3.748	0.623-22.536	0.149
CRP	0.000	1.000	0.995-1.005	0.864
PCT	-0.003	0.997	0.979-1.017	0.792
At Day 7 after admission (Day7)				
GPR174	-5.557	0.004	0.001-0.150	0.003
CRP	0.010	1.010	1.001-1.020	0.031
PCT	0.079	1.082	0.969-1.209	0.162

Adjusted by age and gender.

OR, odds ratio; 95% CI, 95% confidence interval CRP, C-reactive protein; PCT, procalcitonin.

**Figure 4:**
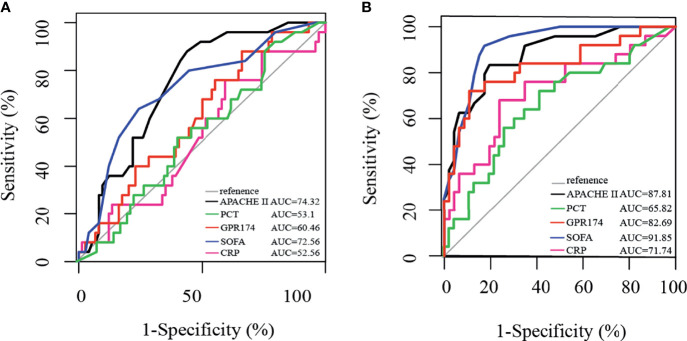
Receiving operating characteristic (ROC) curve for predicting 90-day mortality in septic patients. **(A)** ROC of APACHE II score, PCT, *GPR174* mRNA, SOFA score, and CRP for mortality at ICU admission. **(B)** ROC of APACHE II score, PCT, *GPR174* mRNA, SOFA score, and CRP for mortality at Day 7 after ICU admission.

We further investigated the prognostic value of *GPR174* mRNA in sepsis with the ROC analysis. The area under the ROC curve (AUC) of *GPR174* mRNA at D7 was higher than that at D1 (0.83 vs. 0.60; *P* < 0.001). Furthermore, *via* comparing the AUC of *GPR174* mRNA with APACHE II score, SOFA score, CRP, PCT at D7, we found that the AUC of *GPR174* mRNA was higher than that of PCT (0.83 vs. 0.66; *P* = 0.05). However, there was no difference when compared with CRP (AUC = 0.72, *P* = 0.21), APACHE II score (AUC = 0.88; *P* = 0.704), and SOFA score (AUC = 0.92; *P* = 0.26) (**Figure 4**).

### The Clinical Value of *GPR174* mRNA at Day 7 in Predicting the 90-Day Mortality

**Figure 5:**
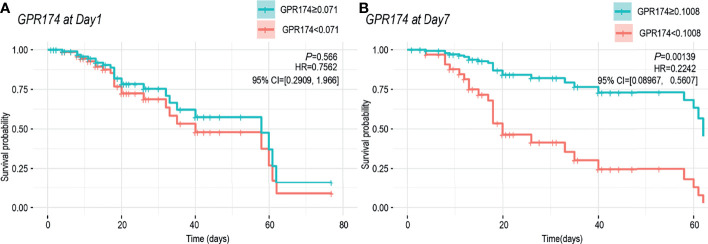
Cox regression model for survival analysis. **(A)** K-M survival curve of the *GPR174* mRNA expression at ICU admission. Patients were divided into higher and lower *GPR174* mRNA groups according to the cut-off level of 0.071. **(B)** K-M survival curve of the *GPR174* mRNA expression at D7 after ICU admission. Patients were divided into higher and lower *GPR174* mRNA groups according to the cut-off level of 0.1008. HR: Hazard Ratio. 95% CI: 95% confidence interval.

The samples were divided into higher and lower *GPR174* mRNA groups according to ROC cut-off point and the K-M survival curve was obtained by Cox regression analysis. At D1, the cut-off level for *GPR174* mRNA is 0.071, and the sensitivity and specificity were 0.880 and 0.338, respectively. At D7, the cut-off level for *GPR174* mRNA is 0.101, and the sensitivity and specificity were 0.890 and 0.720, respectively. The Kaplan-Meier curves showed that no significant difference was found between the two groups at D1 [HR = 0.756, 95% CI = (0.291, 1.966), *P* = 0.566], while the mortality of the lower *GPR174* mRNA group at D7 was significantly higher than that of the higher *GPR174* mRNA group [HR = 0.224, 95% CI = (0.090, 0.561), *P* = 0.001] (**Figure 5**).

### The Dynamic Changes of *Gpr174* mRNA in Mice After CLP-Induced Sepsis

To further study the expression of *Gpr174* mRNA in sepsis, a CLP-induced septic mice model (n = 20 per group) was used to test the expression of *Gpr174* mRNA in PBMC and spleen. Compared to control mice with sham surgery, the levels of *Gpr174* mRNA in PBMC significantly decreased in septic mice, then met an ascending inflection point at 72 h (**Supplementary Figure 3A**), while it continuously decreased in the spleen (**Supplementary Figure 3B**).

The pathological injury of the liver and lung after CLP was confirmed by H&E staining (n = 5 per group). The results showed that clear polygonal hepatic lobules with obvious hepatic cords and sinuses were seen in the liver in the sham surgery group. However, the boundaries of some hepatic lobules were not clear after CLP, and hepatic cord disorder could be seen in hepatic lobules. The damage was most severe at 72 h, which was characterized by cell edema, infiltration of inflammatory cells, and hepatic sinus congestion (**Supplementary Figure 3C**). As for lung tissues, the alveolar cavity was clearly visible in the sham surgery group, while most of the alveolar cavity showed exudation and edema. Severe inflammatory cells infiltration was observed at 72 h after CLP (**Supplementary Figure 3D**).

### *Gpr174*-Deficiency Alleviated Mortality and Inflammatory Response in CLP-Induced Septic Mice

Compared with sham mice, the mortality rate of *Gpr174*-KO septic mice was dramatically decreased from 55% to 25% (n = 20 per group) (**Supplementary Figure 4A**). In addition, *Gpr174*-deficiency significantly alleviated the pathology scores of lung and liver, respectively. H&E staining showed that inflammatory cell infiltration and edema of lung tissue were less severe in *Gpr174*-KO + CLP mice compared to WT + CLP mice (**Supplementary Figure 4B**). Moreover, the hepatic cells edema was less in *Gpr174*-KO + CLP mice than that in WT + CLP mice (**Supplementary Figure 4C**).

To evaluate the effect of GPR174 on the dysregulated systemic inflammation, the serum levels of IL-1β, TNF-α, and IL-10 were examined at 24 h after the CLP challenge (n = 5 per group). The results showed that the levels of IL-1β and TNF-α were significantly decreased in *Gpr174*-KO+CLP mice than in the WT+CLP mice, and the levels of IL-10 were significantly increased (**Supplementary Figure 5**).

**Figure 6:**
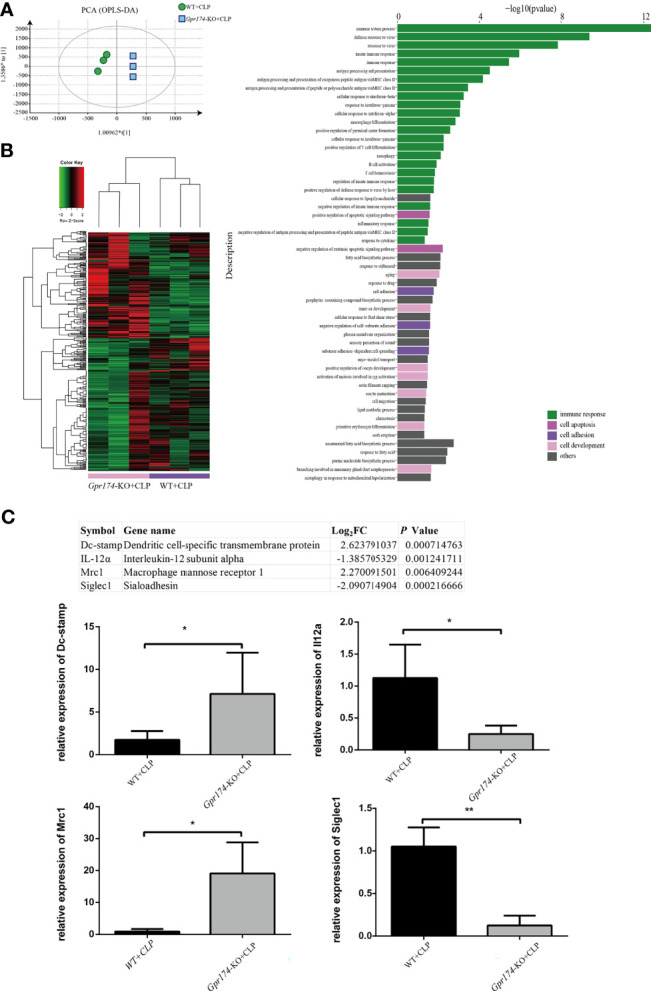
Distinct transcriptional signature in spleen after CLP treated between Gpr174-KO and wild type mice (n = 3 per group). **(A)** Sample correlation was computed by PCA analysis. Percentages in PCA analysis axis indicated the proportional variance explained by PC. **(B)** Differentially expression genes (DEG) were expressed by heatmap with normalized raw z-scores (left) and pyramid plot with -log_10_ (*P* value) demonstrated the involved pathways form DES genes (right). **(C)** qPCR verification of most differentially expressed genes (n = 3-6). Representative genes from the table were verified by qPCR. Values are mean with SEM, ^*^P < 0.05, ^**^P < 0.01.

Then, RNA-seq was used to explore the mechanism of GPR174 in the pathogenesis of sepsis. A total of 10,389 genes were identified compared between *Gpr174*-KO mice and WT mice at 24 h after the CLP challenge (n = 3 per group). *Gpr174*-KO+CLP mice and WT+CLP mice were well separated by cluster PCA analysis (**Figure 6A**). We defined the identified genes as differentially expressed genes (DEPs) if there was a log2FC in excess of 1.5 or less than -1.5, FDR < 0.05. A total of 360 genes changed significantly as shown in the heatmap (**Figure 6B**). GO analysis and Kyoto Encyclopedia of Genes and Genomes (KEGG) pathway enrichment analysis were performed to provide a further understanding of these results. A similar differential effect was observed in the molecular and immunologic pathways that were impacted between *Gpr174*-KO+CLP mice and WT+CLP mice. Further validation of the findings was carried out by qPCR. Intriguingly, we found that representative potential mediators of the immune response, including dendritic cell-specific transmembrane protein and macrophage mannose receptor 1 were highly upregulated, while interleukin-12 subunit alpha and Sialoadhesin were downregulated (**Figure 6C**).

## Discussion

Early diagnosis and intervention are important for improving the prognosis of sepsis. Although several serum biomarkers, APACHE II and SOFA scores were applied to diagnose and assess the illness severity of sepsis in clinical practice (21), there were no ideal targeted biomarkers to predict the initiate and progress of sepsis. Recently, GPR174, a seven-transmembrane G protein-coupled receptor, was identified to be mainly expressed on immune cells such as T/B cells (22) and reported as a risk factor for subcutaneous metastases (23), Graves’ disease (24), and autoimmune Addison’s disease (25). To explore the effects of GPR174 in sepsis, we conducted a series of animal studies and found that GPR174 could regulate the anti-inflammatory response by negatively regulating Treg and B cell functions and attenuating the tissue injury (18, 26). However, the association between GPR174 and severity, mortality of septic patients was still unknown.

In this prospective observational study, we found that the relative expression of *GPR174* mRNA was significantly lower in septic patients than that in non-septic ICU controls and healthy volunteers at D1, which indicated that GPR174 might be a novel biomarker for early diagnosis of sepsis. Moreover, decreased relative expression of serum *GPR174* mRNA was related to the illness severity of sepsis. Importantly, both logistic regression and Cox regression analysis showed that GPR174 was an independent predictor of 90-day mortality in septic patients. Similarly, declining expression of *GPR174* mRNA was found in CLP-induced septic mice. All these results suggested that decreasing *GPR174* mRNA was associated with increased mortality in sepsis. We further carried out the change of the markers on individuals dynamically, including *GPR174* mRNA, APACHE II and SOFA scores, CRP, and PCT. Interestingly, only *GPR174* mRNA showed remarkable differences both in the non-survivor and survivor groups (ascending in the survivors and descending in non-survivors, respectively), which indicated that GPR174 might be a sensitivity prognostic biomarker in sepsis.

During severe infection, the body could eliminate pathogens *via* activating inflammatory reaction, which in turn leads to tissue damage due to uncontrolled cytokine storm (27–29). In view of the role of GPR174 in immune response, we focused on the relationship between GPR174 and immune indicators such as IL-2R, IL-6, IL-8, IL-10, and the counts of WBC, neutrophil, and lymphocyte in septic patients. *GPR174* mRNA had a positive correlation with the counts of lymphocytes. In addition, transcriptomic results showed a shift in *Gpr174*-KO mice, involving T cell homeostasis after the CLP challenge. Meanwhile, the lack of *GPR174* had significantly decreased the serum concentrations of proinflammatory cytokines (IL-1β and TNF-α) after the CLP challenge in mice, while increased the serum concentration of anti-inflammatory cytokines (IL-10). Following re-exposure to LPS, macrophages exhibit an immunosuppressive state known as LPS tolerance, which is characterized by repressed proinflammatory cytokine production. Recently, Chiara Porta et al. reported that LPS-tolerant macrophages have the phenotype of M2-polarized cells (30). M2 macrophages produce anti-inflammatory cytokines/chemokines such as IL-10, TGF-β, and CCL18 (AMAC-1). IL-10 has been shown to be expressed in LPS-resistant macrophages and limits excessive inflammatory reactions in response to endotoxin (31). Here, we previously reported a *Gpr174*-efficiently Treg that could promote polarization of macrophages toward anti-inflammatory M2 macrophages by IL-10 and cell-contact pathway both *in vitro* and *in vivo* (18). These all indicated that the GPR174 might be involved in LPS tolerance.

Other potential mechanisms in sepsis include the decreased expression of a broad array of downregulation of numerous positive costimulatory molecules, and upregulation of inhibitory receptors/ligands (32–34). Specifically, transcripts of Dc-stamp, which is involved in regulating dendritic cell antigen presentation activity and played a role in the maintenance of immune self-tolerance (35), were highly upregulated in *Gpr174*-KO mice. Macrophage mannose receptor 1 (Mrc1), known as a phagocytic receptor for bacteria, fungi, and other pathogens (36, 37), was also upregulated in *Gpr174*-KO mice. On the other hand, Interleukin-12 subunit alpha (IL-12α), acting as a growth factor of activated T and NK cells, had obviously decreased in the present transcriptomic results (38). Similarly, Sialoadhesin (Siglec1), which depending on the IFN/JAK/STAT1 signaling pathway (39), had obviously decreased in *Gpr174*-KO mouse vs. wild-type mouse after CLP injury. These results revealed and supported the potential role of GPR174 on immunoregulation, however, the specific mechanism remains to be studied.

Several limitations should be addressed in the current study. First, the small sample size did not allow in-depth analysis of the relationships between GPR174 and disease severity, as well as mortality, so a larger multicenter study is required in the future. Second, further studies are needed to explore the exact function and mechanism of GPR174 in the host immune response during sepsis.

In conclusion, our results first showed that the expression of *GPR174* mRNA is associated with disease severity and mortality in sepsis. Monitoring the levels of *GPR174* mRNA could be effective in the identification of septic patients at high risk of death. Further studies are needed to explore the regulating mechanism of GPR174 on immune cells during sepsis.

## Data Availability Statement

The datasets presented in this study can be found in online repositories. The names of the repository/repositories and accession number(s) can be found below: https://www.ncbi.nlm.nih.gov/, BioProject ID: PRJNA771765.

## Ethics Statement

The studies involving human participants were reviewed and approved by the Ethics Committee Study Board of Zhongshan Hospital, Fudan University, Shanghai, China. The patients/participants provided their written informed consent to participate in this study. The animal study was reviewed and approved by the Ethics Committee Study Board of Zhongshan Hospital, Fudan University, Shanghai, China.

## Author Contributions

ZS, YY, and CT conceived and designed the experiments. YH and ZK collected the samples and clinical data. JW performed the animal experiments. YC and LX performed the statistical analysis. WW and MX contributed reagents/materials/analysis tools. SM helped to draft and revise the manuscript. All authors contributed to the article and approved the submitted version.

## Funding

This work was supported by Shanghai Municipal Health Bureau (Grant No. ZXYXZ-201906, Grant No. GWV-10.2-XD04), Science and Technology of Shanghai Committee (Grant No. 20Y11900100, Grant No. 21MC1930400, Grant No.20DZ 2261200), and National Natural Science Foundation of China (Grant No. 82072214).

## Conflict of Interest

The authors declare that the research was conducted in the absence of any commercial or financial relationships that could be construed as a potential conflict of interest.

## Publisher’s Note

All claims expressed in this article are solely those of the authors and do not necessarily represent those of their affiliated organizations, or those of the publisher, the editors and the reviewers. Any product that may be evaluated in this article, or claim that may be made by its manufacturer, is not guaranteed or endorsed by the publisher.

## Supplementary Material

Supplementary Figure 1 Study flowchart.

Supplementary Figure 2 The trend of the markers on individuals from D1 to D7 in septic patients. The individual trend of GPR174 mRNA, APACHE II score, SOFA score, CRP, IL-2R, PCT, IL-6, IL-8, and IL-10, the counts of Neu, Lym, and WBC were tested both in non-survivor and survivor of septic patients, respectively. Dots represent individual participants. P values less than 0.05 were considered statistically significant.

Supplementary Figure 3 The changes of Gpr174 mRNA and injury of vital organs in CLP-induced septic mouse. **(A)** Levels of *Gpr174* mRNA in PBMC were tested by quantitative PCR, which were collected at 0 h, 12 h, 24 h, 72 h, 1 w, 2 w in CLP-induced sepsis. **(B)** Levels of *Gpr174* mRNA in the spleen were tested by quantitative PCR collected at 0 h, 12 h, 24 h, 72 h, 1 w, 2 w in CLP-induced sepsis. **(C)** Representative examples of hematoxylin and eosin (H&E)-stained liver tissues from mice at 0 h, 12 h, 24 h, 1 w, 2 w after CLP (n = 5 per group). Hepatic cord disorder (shown by the black arrow) could be seen in hepatic lobules in septic mice. **(D)** Representative examples of H&E-stained lung tissues from mice at 0 h, 12 h, 24 h, 1 w, 2 w after CLP (n = 5 per group). Alveolar cavity showed exudation, edema, and hemorrhage by the black arrow. **(E, F)** Histological scores of the liver and lung in CLP-induced septic mice (n = 5 per group). *P <0.05; **P <0.01.

Supplementary Figure 4 The effect of *Gpr174* deficiency on CLP-induced sepsis (n = 20 per group). **(A)** Survival rates were monitored for 1 w in *Gpr174*-KO mice compared with wild type (WT) after CLP-induced sepsis (n = 20 per group). **(B)** Representative H&E staining examples and histological scores for lung tissues at 24 h after CLP-induced sepsis. **(C)** Representative H&E staining examples and histological scores for liver tissues at 24 h after CLP (n = 5 per group). **P* < 0.05; ***P* < 0.01.

Supplementary Figure 5
*Gpr174* regulated the production of pro- and anti-inflammatory cytokines in CLP-induced septic mice (n = 5 per group). Cytokines in blood from septic mice were determined by ELISA at 24 h after CLP. **(A, C)** IL-1β and TNF-α levels were downregulated in *Gpr174*-KO + CLP mice. **(B)** IL-10 was upregulated compared to WT + CLP group. **P* < 0.05; ***P* < 0.01.

## References

[1] Singer M Deutschman CS Seymour CW Shankar-Hari M Annane D Bauer M. The Third International Consensus Definitions for Sepsis and Septic Shock (Sepsis-3). JAMA (2016) 315:801–10. doi: 10.1001/jama.2016.0287 PMC4968574 26903338

[2] Reinhart K Daniels R Kissoon N Machado FR Schachter RD Finfer S. Recognizing Sepsis as a Global Health Priority- a WHO Resolution. N Engl J Med (2017) 377:414–7. doi: 10.1056/NEJMp1707170 28658587

[3] van der Poll T Van de Veerdonk FL Scicluna BP Netea MG. The Immunopathology of Sepsis and Potential Therapeutic Targets. Nat Rev Immunol (2017) 17(7):407–20. doi: 10.1038/nri.2017.36 28436424

[4] Liu Q Yao Y-M. Inflammatory Response and Immune Regulation of High Mobility Group Box-1 Protein in Treatment of Sepsis. World J Emerg Med (2010) 1(2):93–8. PMC4129755 25214948

[5] Blaurock-Möller N Gröger M Siwczak F Dinger J Schmerler D Mosig AS. CAAP48, A New Sepsis Biomarker, Induces Hepatic Dysfunction in an Invitroliver-on-Chip Model. Front Immunol (2019) 10:273. doi: 10.3389/fimmu.2019.00273 30873161 PMC6401602

[6] Liu S Wang X She F Zhang W Liu H Zhao X. Effects of Neutrophil-To-Lymphocyte Ratio Combined With Interleukin-6 in Predicting 28-Day Mortality in Patients With Sepsis. Front Immunol (2021) 12:639735. doi: 10.3389/fimmu.2021.639735 33796105 PMC8007868

[7] Zhu T Su Q Wang C Shen L Chen H Feng S. SDF4 Is a Prognostic Factor for 28-Days Mortality in Patients With Sepsis *via* Negatively Regulating ER Stress. Front Immunol (2021) 12:659193. doi: 10.3389/fimmu.2021.659193 34326834 PMC8313857

[8] Trzaskowski B Latek D Yuan S Ghoshdastider U Debinski A Filipek S. Action of Molecular Switches in GPCRs–theoretical and Experimental Studies. Curr Med Chem (2012) 19(8):1090–109. doi: 10.2174/092986712799320556 PMC3343417 22300046

[9] Wettschureck N Offermanns S. Mammalian G Proteins and Their Cell Type Specific Functions. Physiol Rev (2005) 85(4):1159–204. doi: 10.1152/physrev.00003.2005 16183910

[10] Garcia-Marcos M. Complementary Biosensors Reveal Different G-Protein Signaling Modes Triggered by GPCRs and Non-Receptor Activators. Elife (2021) 10:e65620. doi: 10.7554/eLife.65620 33787494 PMC8034979

[11] Inoue A Ishiguro J Kitamura H Arima N Okutani M Shuto A. TGF-Alpha Shedding Assay: An Accurate and Versatile Method Fordetecting GPCR Activation. Nat Methods (2012) 9:1021–9. doi: 10.1038/nmeth.2172 22983457

[12] Konkel JE Zhang D Zanvit P Chia C Zangarle-Murray T Jin W. Transforming Growth Factor-β Signaling in Regulatory T Cells Controls T Helper-17 Cells and Tissue-Specific Immune Responses. Immunity (2017) 46(4):660–74. doi: 10.1016/j.immuni.2017.03.015 PMC12230991 28423340

[13] Zhao R Chen X Ma W Zhang J Guo J Zhong X. A GPR174–CCL21 Module Imparts Sexual Dimorphism to Humoral Immunity. Nature (2020) 577(7790):416–20. doi: 10.1038/s41586-019-1873-0 31875850

[14] Sugita K Yamamura C Tabata K Fujita N. Expression of Orphan G-Protein Coupled Receptor GPR174 in CHO Cells Induced Morphological Changes and Proliferation Delay *via* Increasing Intracellular cAMP. Biochem Biophys Res Commun (2013) 430(1):190–5. doi: 10.1016/j.bbrc.2012.11.046 23178570

[15] Barnes MJ Li CM Xu Y An J Huang Y Cyster JG. The Lysophosphatidylserine Receptor GPR174 Constrains Regulatory T Cell Development and Function. J Exp Med (2015) 212:1011–20. doi: 10.1084/jem.20141827 PMC4493414 26077720

[16] Yue J Zhao X. GPR174 Suppression Attenuates Retinopathy in Angiotensin II (Ang II)-Treated Mice by Reducing Inflammation *via* PI3K/AKT Signaling. BioMed Pharmacother (2020) 122:109701. doi: 10.1016/j.biopha.2019.109701 31918274

[17] Chu X Shen M Xie F Miao XJ Shou WH Liu L. An X Chromosome-Wide Association Analysis Identifies Variants in GPR174 as a Risk Factor for Graves’ Disease. J Med Genet (2013) 50:479–85. doi: 10.1136/jmedgenet-2013-101595 PMC3686253 23667180

[18] Qiu D Chu X Hua L Yang Y Li K Han Y. Gpr174-Deficient Regulatory T Cells Decrease Cytokine Storm in Septic Mice. Cell Death Dis (2019) 10(3):233–47. doi: 10.1038/s41419-019-1462-z PMC6408576 30850582

[19] Rittirsch D Huber-Lang MS Flierl MA Ward PA. Immunodesign of Experimental Sepsis by Cecal Ligation and Puncture. Nat Protoc (2009) 4:31–6. doi: 10.1038/nprot.2008.214 PMC2754226 19131954

[20] Robin X Turck N Hainard A Tiberti N Lisacek F Sanchez JC. pROC: An Open-Source Package for R and S+ to Analyze and Compare ROC Curves. BMC Bioinf (2021) 7:77. doi: 10.1186/1471-2105-12-77 PMC3068975 21414208

[21] Sandroni C Nolan J Cavallaro F Antonelli M. In-Hospital Cardiac Arrest: Incidence, Prognosis and Possible Measures to Improve Survival. Intensive Care Med (2007) 33(2):237–45. doi: 10.1007/s00134-006-0326-z 17019558

[22] Makide K Uwamizu A Shinjo Y Ishiguro J Okutani M Inoue A. Novel Lysophosphoplipid Receptors: Their Structure and Function. J Lipid Res (2014) 55(10):1986–95. doi: 10.1194/jlr.R046920 PMC4173991 24891334

[23] Qin Y Verdegaal EME Siderius M Bebelman JP Smit MJ Leurs R. Quantitative Expression Profiling of G-Protein-Coupled Receptors (GPCRs) in Metastatic Melanoma: The Constitutively Active Orphan GPCR GPR18 as Novel Drug Target. Pigment Cell Melanoma Res (2011) 24(1):207–18. doi: 10.1111/j.1755-148X.2010.00781.x 20880198

[24] Zhao S Xue L Liu W Gu ZH Pan CM Yang SY. Robust Evidence for Five New Graves’ Disease Risk Loci From a Staged Genome-Wide Association Analysis. Hum Mol Genet (2013) 22(16):3347–62. doi: 10.1093/hmg/ddt183 23612905

[25] Napier C Mitchell AL Gan E Wilson I Pearce SH. Role of the X-Linked Gene GPR174 in Autoimmune Addison’s Disease. J Clin Endocrinol Metab (2015) 100(1):187–90. doi: 10.1210/jc.2014-2694 25295623

[26] Zhu M Li C Song Z Mu S Wang J Wei W. The Increased Marginal Zone B Cells Attenuates Early Inflammatory Responses During Sepsis in Gpr174 Deficient Mice. Int Immunopharmacol (2020) 81:106034. doi: 10.1016/j.intimp.2019.106034 31786099

[27] Sewnath ME Olszyna DP Birjmohun R ten Kate FJ Gouma DJ van Der Poll T. IL-10-Deficient Mice Demonstrate Multiple Organ Failure and Increased Mortality During Escherichia Coli Peritonitis Despite an Accelerated Bacterial Clearance. J Immunol (2001) 166:6323–31. doi: 10.4049/jimmunol.166.10.6323 11342656

[28] Matsukawa A Takeda K Kudo S Maeda T Kagayama M Akira S. Aberrant Inflammation and Lethality to Septic Peritonitis in Mice Lacking STAT3 in Macrophages and Neutrophils. J Immunol (2003) 171:6198–205. doi: 10.4049/jimmunol.171.11.6198 14634136

[29] Liu G Burns S Huang G Boyd K Proia RL Flavell RA. The Receptor S1P1 Overrides Regulatory T Cell-Mediated Immune Suppression Through Akt-mTOR. Nat Immunol (2009) 10:769–77. doi: 10.1038/ni.1743 PMC2732340 19483717

[30] Porta C Rimoldi M Raes G Brys L Ghezzi P Di Liberto D. Tolerance and M2 (Alternative) Macrophage Polarization Are Related Processes Orchestrated by P50 Nuclear Factor kappaB. Proc Natl Acad Sci USA (2009) 106(35):14978–83. doi: 10.1073/pnas.0809784106 PMC2736429 19706447

[31] Grütz G. New Insights Into the Molecular Mechanism of Interleukin-10-Mediated Immunosuppression. J Leukoc Biol (2005) 77(1):3–15. doi: 10.1189/jlb.0904484 15522916

[32] Davenport EE Burnham KL Radhakrishnan J Humburg P Hutton P Mills TC. Genomic Landscape of the Individual Host Response and Outcomes in Sepsis: A Prospective Cohort Study. Lancet Respir (2016) 4:259–71. doi: 10.1016/S2213-2600(16)00046-1 PMC4820667 26917434

[33] Thampy LK Remy KE Walton AH Hong Z Liu K Liu R. Restoration of T Cell Function in Multi-Drug Resistant Bacterial Sepsis After Interleukin-7, Anti-PD-L1, and OX-40 Administration. PloS One (2018) 13:e0199497. doi: 10.1371/journal.pone.0199497 29944697 PMC6019671

[34] Dobin A Davis CA Schlesinger F Drenkow J Zaleski C Jha S. STAR: Ultrafast Universal RNA-Seq Aligner. Bioinformatics (2013) 29:15–21. doi: 10.1093/bioinformatics/bts635 23104886 PMC3530905

[35] Cardoso CC Pereira AC de Sales Marques C Moraes MO. Leprosy Susceptibility: Genetic Variations Regulate Innate and Adaptive Immunity, and Disease Outcome. Future Microbiol (2011) 6(5):533–49. doi: 10.2217/fmb.11.39 21585261

[36] Gaudet P Livstone MS Lewis SE Thomas PD. Phylogenetic-Based Propagation of Functional Annotations Within the Gene Ontology Consortium. Brief Bioinform (2011) 12(5):449–62. doi: 10.1093/bib/bbr042 PMC3178059 21873635

[37] Zhou Y Do DC Ishmael FT Squadrito ML Tang HM Tang HL. Mannose Receptor Modulates Macrophage Polarization and Allergic Inflammation Through miR-511-3p. Allergy Clin Immunol (2018) 141(1):350–64. doi: 10.1016/j.jaci.2017.04.049 PMC5944850 28629744

[38] Ruffell B Chang-Strachan D Chan V Rosenbusch A Ho CM Pryer N. Macrophage IL-10 Blocks CD8+ T Cell-Dependent Responses to Chemotherapy by Suppressing IL-12 Expression in Intratumoral Dendritic Cells. Cancer Cell (2014) 26(5):623–37. doi: 10.1016/j.ccell.2014.09.006 PMC4254570 25446896

[39] Zheng Q Hou J Zhou Y Yang Y Xie B Cao X. Siglec1 Suppresses Antiviral Innate Immune Response by Inducing TBK1 Degradation *via* the Ubiquitin Ligase TRIM27. Cell Res (2015) 25(10):1121–36. doi: 10.1038/cr.2015.108 PMC4650625 26358190

