# Supplementary figures and images for: GPR174 mRNA Acts as a Novel Prognostic Biomarker for Patients With Sepsis via Regulating the Inflammatory Response
Source: Front Immunol. 2022 Jan 31;12:789141. doi: 10.3389/fimmu.2021.789141 (PMC8841418; doi:10.3389/fimmu.2021.789141)

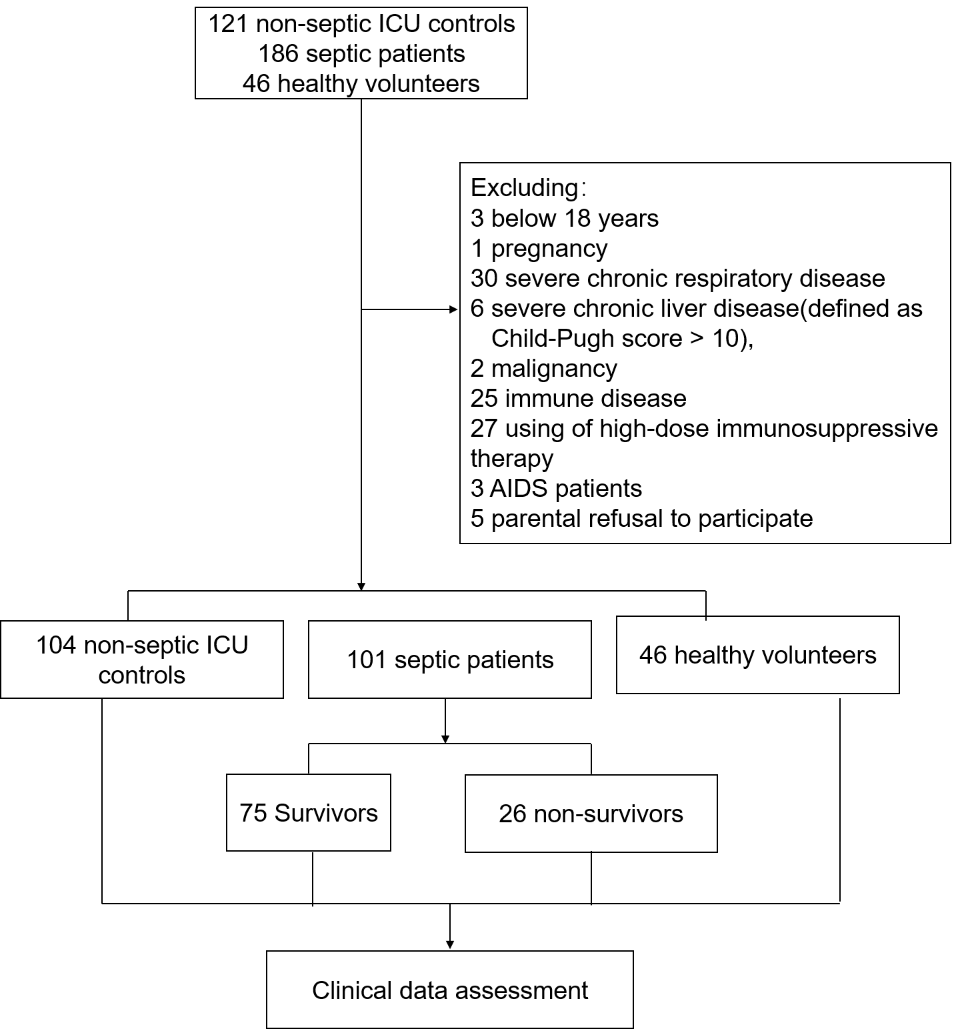

Supplement: Supplementary Figure 1 — Study flowchart. [file Image_1.tif]

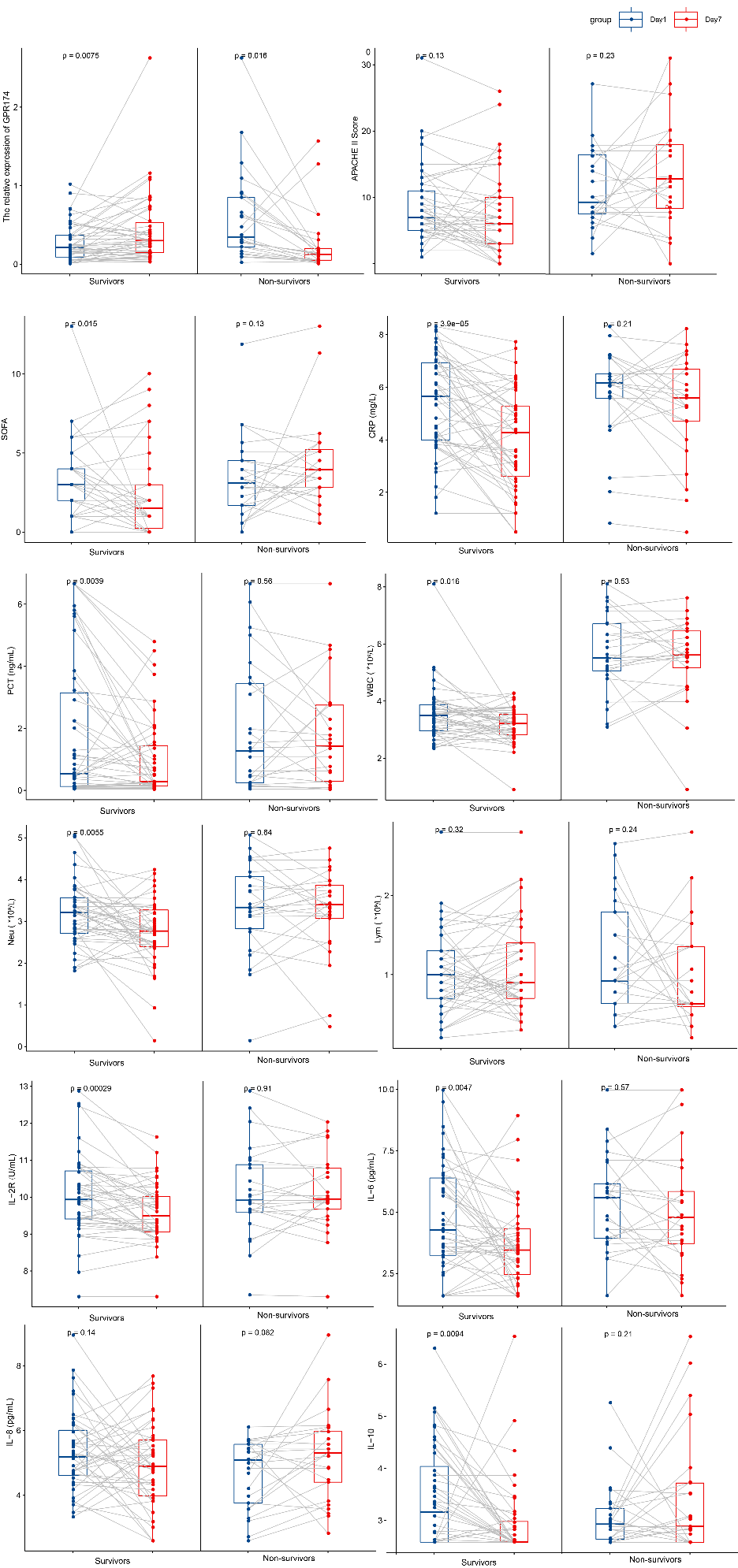

Supplement: Supplementary Figure 2 — The trend of the markers on individuals from D1 to D7 in septic patients. The individual trend of GPR174 mRNA, APACHE II score, SOFA score, CRP, IL-2R, PCT, IL-6, IL-8, and IL-10, the counts of Neu, Lym, and WBC were tested both in non-survivor and survivor of septic patients, respectively. Dots represent individual participants. P values less than 0.05 were considered statistically significant. [file Image_2.tif]

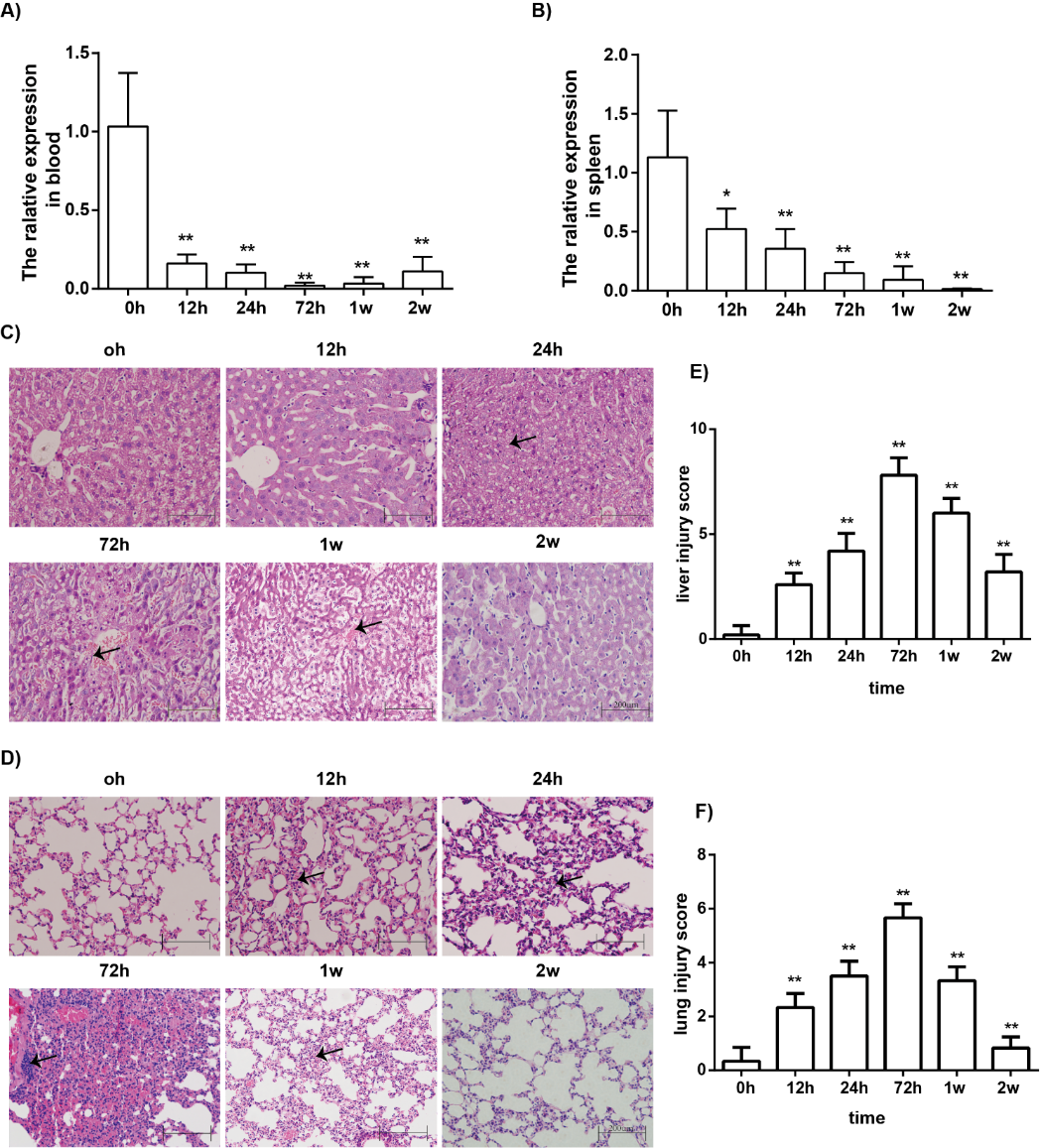

Supplement: Supplementary Figure 3 — The changes of Gpr174 mRNA and injury of vital organs in CLP-induced septic mouse. (A) Levels of Gpr174 mRNA in PBMC were tested by quantitative PCR, which were collected at 0 h, 12 h, 24 h, 72 h, 1 w, 2 w in CLP-induced sepsis. (B) Levels of Gpr174 mRNA in the spleen were tested by quantitative PCR collected at 0 h, 12 h, 24 h, 72 h, 1 w, 2 w in CLP-induced sepsis. (C) Representative examples of hematoxylin and eosin (H&E)-stained liver tissues from mice at 0 h, 12 h, 24 h, 1 w, 2 w after CLP (n = 5 per group). Hepatic cord disorder (shown by the black arrow) could be seen in hepatic lobules in septic mice. (D) Representative examples of H&E-stained lung tissues from mice at 0 h, 12 h, 24 h, 1 w, 2 w after CLP (n = 5 per group). Alveolar cavity showed exudation, edema, and hemorrhage by the black arrow. (E, F) Histological scores of the liver and lung in CLP-induced septic mice (n = 5 per group). *P <0.05; **P <0.01. [file Image_3.tif]

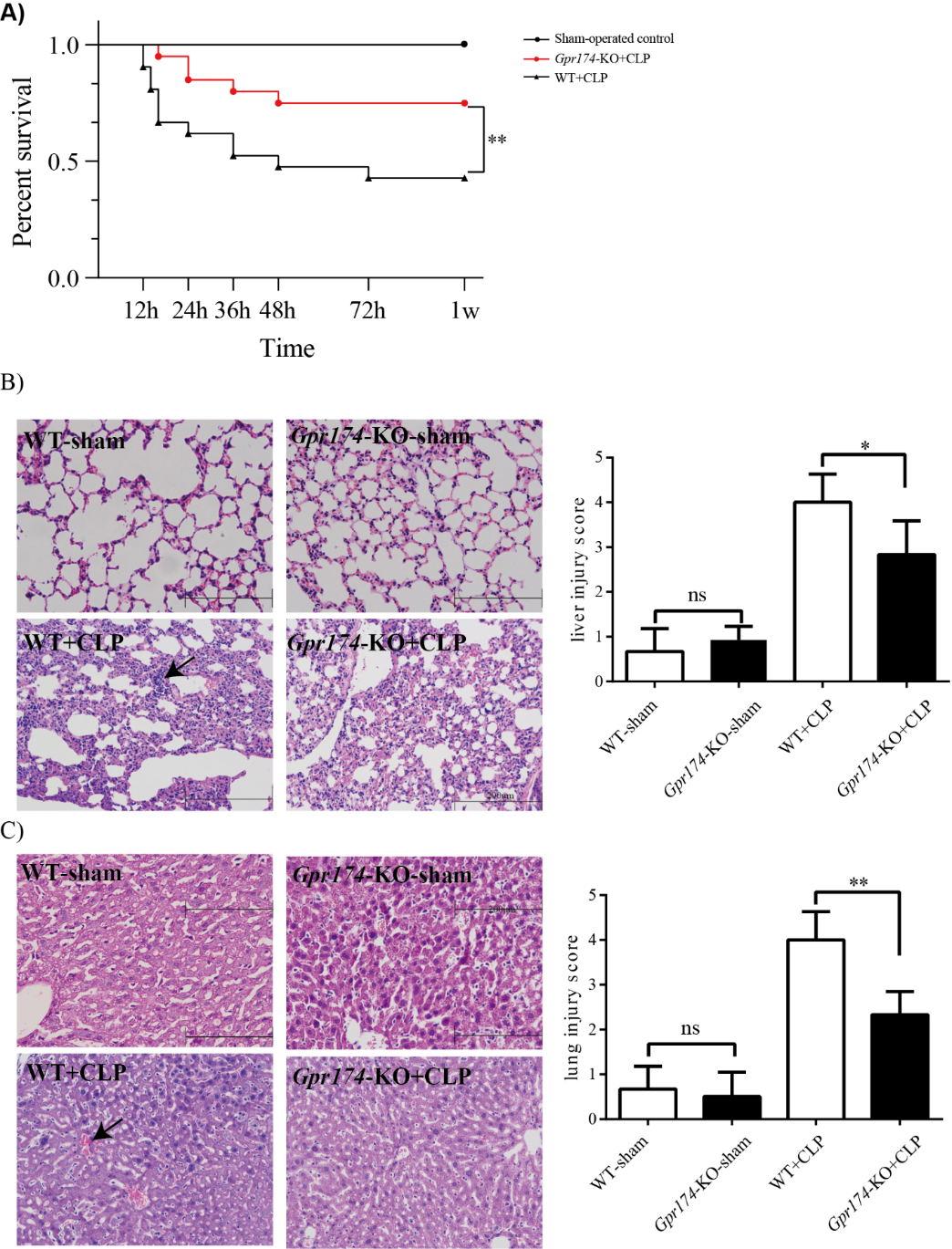

Supplement: Supplementary Figure 4 — The effect of Gpr174 deficiency on CLP-induced sepsis (n = 20 per group). (A) Survival rates were monitored for 1 w in Gpr174-KO mice compared with wild type (WT) after CLP-induced sepsis (n = 20 per group). (B) Representative H&E staining examples and histological scores for lung tissues at 24 h after CLP-induced sepsis. (C) Representative H&E staining examples and histological scores for liver tissues at 24 h after CLP (n = 5 per group). *P < 0.05; **P < 0.01. [file Image_4.tif]

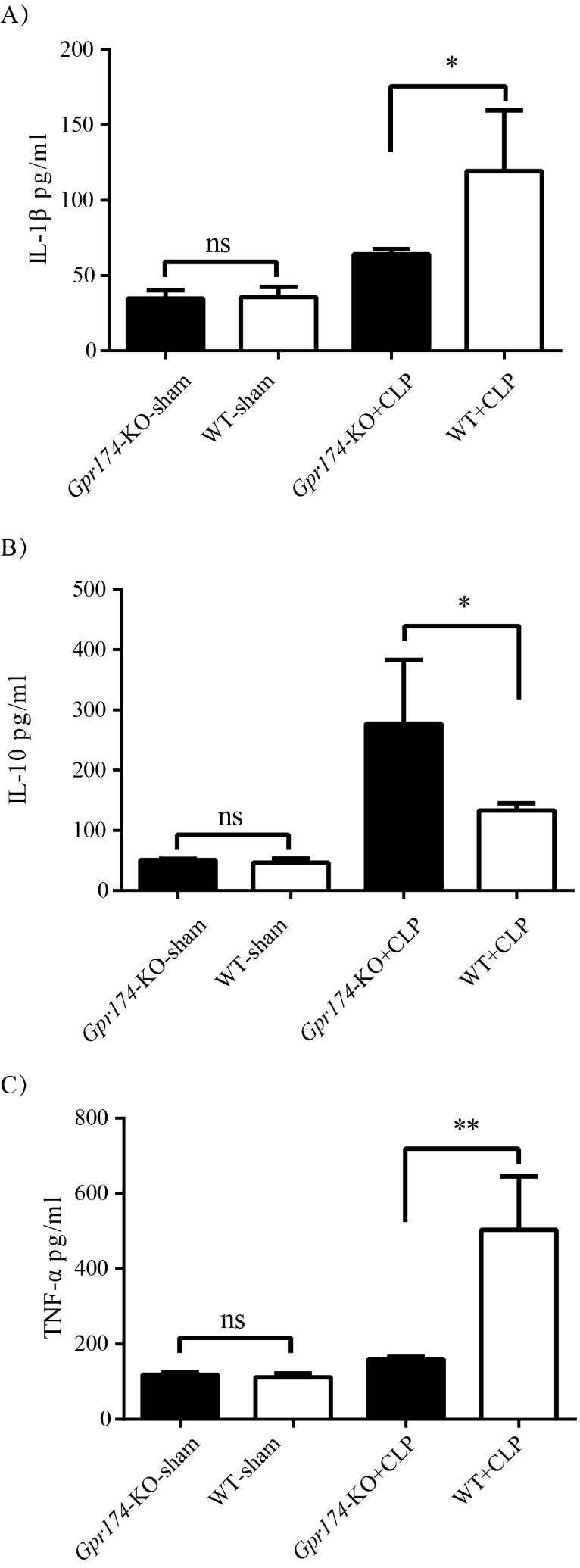

Supplement: Supplementary Figure 5 — Gpr174 regulated the production of pro- and anti-inflammatory cytokines in CLP-induced septic mice (n = 5 per group). Cytokines in blood from septic mice were determined by ELISA at 24 h after CLP. (A, C) IL-1β and TNF-α levels were downregulated in Gpr174-KO + CLP mice. (B) IL-10 was upregulated compared to WT + CLP group. *P < 0.05; **P < 0.01. [file Image_5.tif]
